# Supplementary material for: Hydrodynamic spin-orbit coupling in asynchronous optically driven micro-rotors
Source: Nat Commun. 2023 Jul 11;14:4114. doi: 10.1038/s41467-023-39582-3 (PMC10336088; doi:10.1038/s41467-023-39582-3)
Supplement: Supplementary file 1 — Supplementary Information [file 41467_2023_39582_MOESM1_ESM.pdf]

Supplementary Information for

Hydrodynamic spin-orbit coupling in asynchronous optically driven micro-rotors

Alvin Modin[1,2,4], Matan Yah Ben Zion[1,3,4,⋆], and Paul M Chaikin[1]

[1]Center for Soft Matter Research, Department of Physics, New York University 726 Broadway Avenue, New York, NY 10003, USA

[2]Department of Physics and Astronomy, Johns Hopkins University, Baltimore, Maryland 21218, USA

[3]School of Physics and Astronomy, and the Center for Physics and Chemistry of Living Systems, Tel Aviv University, Tel Aviv 6997801, Israel

[4]These authors contributed equally.

⋆matanbz@gmail.com

Supplementary Notes

Supplementary Note 1: Optical Setup

Our instrument is designed to apply a uniform optical torque field by delivering a collimated beam to the sample plane. A schematic diagram of the setup can be seen in Supplementary Fig. 1a. The experimental system consists of a 3mm infrared (IR) laser, $\lambda = 1064$nm (IPG Photonics, $M^2 = 1$), propagating first through a half-wave plate ($\lambda/2$) followed by a Galilean beam contractor. The telescope can shrink the beam by a factor of 30, while preserving the initial collimation of the laser source. Once the beam diameter has been reduced, the laser propagates through a polarizing beam-splitter (PBS) cube where the S-component of light is reflected upwards. The handedness of the linearly polarized light can be altered via a quarter wave plate ($\lambda/4$) fixed immediately after the PBS atop a precision manual rotation mount.

We image the distribution of the incident near-infrared beam using an infrared viewing card. The pixel intensities of the image are then fit to a Gaussian surface of revolution to obtain an intensity distribution of the beam flux at the sample plane. The flux distribution $J(\boldsymbol{r})$ can be written as

$$J(\boldsymbol{r}) = J_0 \exp\left[-2\frac{\mathbf{r}^2}{\sigma^2}\right] \tag{1}$$

The prefactor corresponds to the flux at the center of the beam and is obtained by integrating the 2-dimensional Gaussian over the area of the beam. Supplementary Fig. 1b shows a typical $J(\boldsymbol{r})$ for $\sigma = 372.8 \pm 5.6\mu$m (FWHM $= 438.9 \pm 6.6\mu$m). We can adjust the flux at the sample by changing the size of the incoming beam, modulating the power at the laser head, or tuning the orientation of the $\lambda/2$- plate.

We illuminate the sample using a 505 nm LED (Thorlabs) to image our photonic rotors. The light emitted by the LED passes through a N-BK7 ground glass diffuser (600 grit) and is then pseudo-collimated with an N-BK7 plano-convex lens (f=25.4mm) that acts as a primary collector lens. After the plano-convex lens, a secondary achromatic doublet lens (f=76.2mm) forms an image of the filament at the position of a field diaphragm. A condenser lens focuses the light at the objective's back focal plane. Particles are imaged using an infinity-corrected Leica HCX PL APO 40x objective (NA=0.85). High-pass index matched IR filters mounted before the camera are used to prevent interference of IR light with the 3.1MP monochrome camera (Imaging Source).

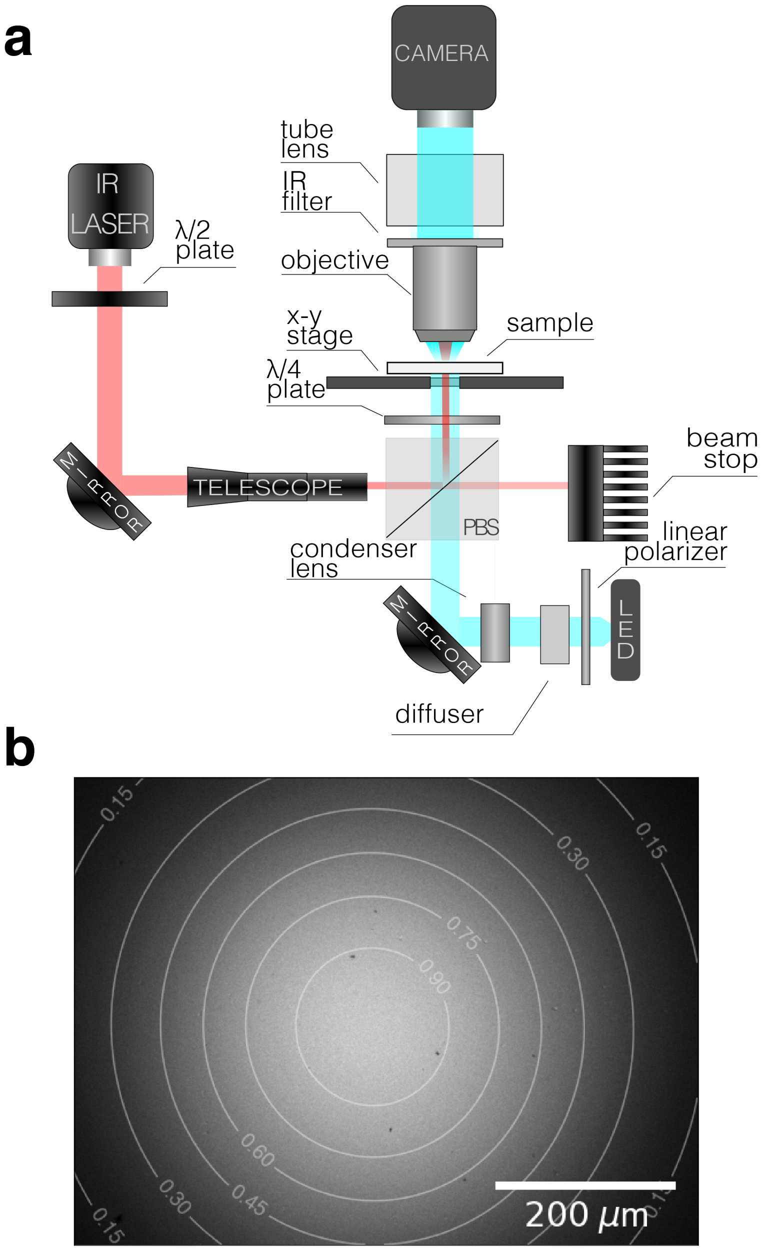

Supplementary Figure 1: **Optical setup used to create a torque field to drive birefringent micro-rotors. a** Detailed schematic of the optical elements used to couple the imaging and laser systems to deliver a defocused beam to the sample plane. **b** Broad field image of the laser beam (contours show relative power given by Gaussian fit). Experiments were performed in a region ∼ 165μm x 125μm, where gradients in the flux are negligible.

Supplementary Note 2: Measuring rotational diffusion using transmission decorrelation through crossed-polarizers

Rotational diffusion is measured by calculating the auto-correlation of the depolarization of the light intensity transmitted through the particles, as measured using cross-polarizers on our custom-built microscope (see Supplementary Fig. 1(a)). Following previous work[1,2], we define the intensity auto-correlation function as $g_{PA}(\tau) = \langle I^*_{PA}(0) I_{PA}(\tau)\rangle / \langle |I_{PA}|^2\rangle$ where I_{PA} is the time-averaged scattered intensity field detected by the camera, and τ is the lag time. To leading order, the translational and rotational dynamics are uncoupled, thus enabling direct measurement of the diffusion matrix $\mathbf{D}_r$ by fitting $g_{PA} = \exp(-6\mathbf{D}_r\tau)$ (Fig. 3 in the main text). In the lubrication limit, diffusive rolling, $D_{r,||}$ (rotation on an axis parallel to the wall) is much slower than diffusive spinning (rotation perpendicular to the wall $D_{r,\perp}$) which dominates the diffusion matrix, $\mathbf{D}_r \approx D_{r,\perp}$[2].

Supplementary Note 3: Measuring the translational diffusion constant of rotors

We record videos of freely-diffusing vaterite micro-spheres and track their instantaneous positions using the Python package TrackPy. TrackPy was downloaded and used without further modification[3]. The package implements the widely used Crocker-Grier particle tracking algorithm[4], which identifies local brightness maxima within an image as candidate particle locations. We track particles diffusing throughout the movie and extract their diffusion constants by computing their mean squared displacements, $\langle \Delta r^2\rangle = 4D_t\tau$ for different lag-times τ. The y-intercept of a linear fit in log directly measures D_t.

Supplementary Note 4: Measuring the reflection coefficient of a vaterite rotor

A: Quantifying the back-scattered radiation by a photonic rotor

Back-scattered light from a photonic rotor generates a radiation pressure that counteracts the gravitational force $F_g = \pi\Delta\rho g d^3/6$ (Supplementary Fig.2). For a given flux J, the average force on a particle with diameter d is given by $\langle F_{rad}\rangle = \frac{RJ\pi d^2}{4c}$. Here c is the speed of light, and R is the reflection coefficient. For vaterite micro-spheres, absorption of IR light is negligible[5]. Micro-spheres travel at a constant velocity through the 100μm capillary when $\langle F_{rad}\rangle > F_g$. This upward motion is balanced by the viscous drag force $F_D = 3\pi\eta d v$. Here v is the average rise velocity of the particle as it travels through a 100μm tall capillary. The procedure for measuring v can be found in the subsection below.

Balancing the forces acting on the particle gives,

$$\frac{RJ_0}{12c\eta} - \frac{1}{18}\frac{\Delta\rho g}{\eta} d = \frac{v}{d}, \tag{2}$$

where the buoyant density $\Delta\rho = 1.43$ g/cc for vaterite particles suspended in D_2O. The velocities for different particle sizes and fluxes are shown in Supplementary Fig. 2. R is extracted by linear fit of Eq. 2 to the data. We find that $R = 0.22 \pm 0.01$ – to our knowledge, this is the first direct measurement of the reflection

coefficient of vaterite micro-spheres and is in good agreement with spherical particles of similar indices of refraction[6].

B: Measuring the average rise velocity v of a particle in the presence of an optical flux

When $\langle F_{\rm rad}\rangle > F_{\rm g}$, particles steadily rise. To monitor the out-of-plane motion of the particles, we first focus on the particles as they freely diffuse in the plane in the presence of no optical flux. Using a translation stage, we vertically shift the imaging focal plane by 100μm, corresponding to the capillary's height where the particles are suspended. We time how long it takes for the particles to re-appear in focus at the top of the capillary. The depth of field of our 40x objective is $\approx 1\mu$m – less than the diameter of the particles – ensuring good accuracy of our measurements.

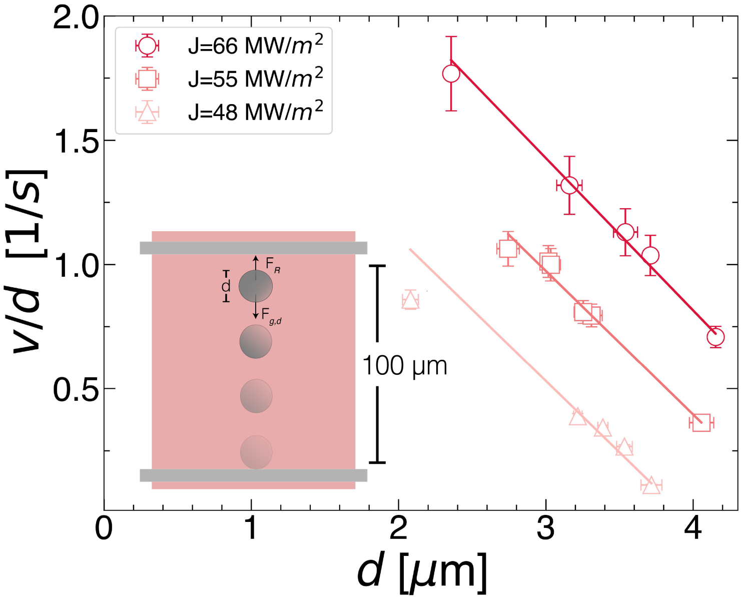

Supplementary Figure 2: **Linear fits to the ratio between the average rise velocity and particle size v/d allows for direct measurement of the reflection coefficient R for vaterite microspheres.** Across three different fluxes, we compute an average $R = 0.22 \pm 0.01$. Buoyant densities obtained from the slope of the lines vary by $< 10\%$ from the expected value. Error bars correspond to the standard deviation.

Supplementary Note 5: Spinning angular frequency Ω of a birefringent particle

In the low-Reynolds number limit, a sphere spinning in a fluid at angular frequency Ω experiences a viscous torque $\tau_{\rm v} = \pi\eta d^3\Omega$, dependant on the viscosity of the fluid η and the particle's diameter, d. $\tau_{\rm v}$ is balanced by the optical torque, $\tau_{\rm p}$, that is responsible for generating the spinning motion. The magnitude of $\tau_{\rm p}$ depends on the incident light's polarization ellipticity ϕ and wavelength λ according to[7],

$$\tau_{\rm p} = \frac{\lambda PT}{2\pi c}\left[\left(1-\cos\frac{2\pi\Delta nt}{\lambda}\right)\sin 2\phi - \sin\left(\frac{2\pi\Delta nt}{\lambda}\right)\cos 2\phi\sin 2\theta\right]. \quad (3)$$

Here, P represents the power incident on the particle, and c is the speed of light. The first term of Eq. 3 corresponds to the "spinning torque"; the argument of cos can be thought of as the degree to which

the particle acts as a phase-retarder, providing the maximum change in the transmitted light's spin-angular momentum when the particle is exactly the thickness of a half-wave plate. Therefore, it must depend on the physical properties of the particle, such as its birefringence Δn, transmissivity T, and thickness t. The second term in Eq. 3 is a torque that seeks to align the fast axis of the particle with the plane of polarization. To derive an equation for the spinning rate in the presence of a circularly polarized incident beam ($\phi = \pi/4$), we approximate the spherical particles as cylinders so that their diameter $d \approx t$[8]. In this limit, the alignment torque vanishes, and the optical torque is proportional to $\Delta\sigma \equiv 1 - \cos\left(\frac{2\pi\Delta n d}{\lambda}\right)$. Rewriting P in terms of the power flux J, $P = J\pi d^2/4$, the optical torque becomes $\tau_{\mathrm{p}} = \Delta\sigma T J \lambda d^2/8c$. Equating τ_{p} and τ_{v} and solving for Ω gives,

$$\Omega = \frac{\mathrm{T J}\lambda}{8\pi c\eta d}\left[1 - \cos\left(\frac{2\pi\Delta n d}{\lambda}\right)\right]. \tag{4}$$

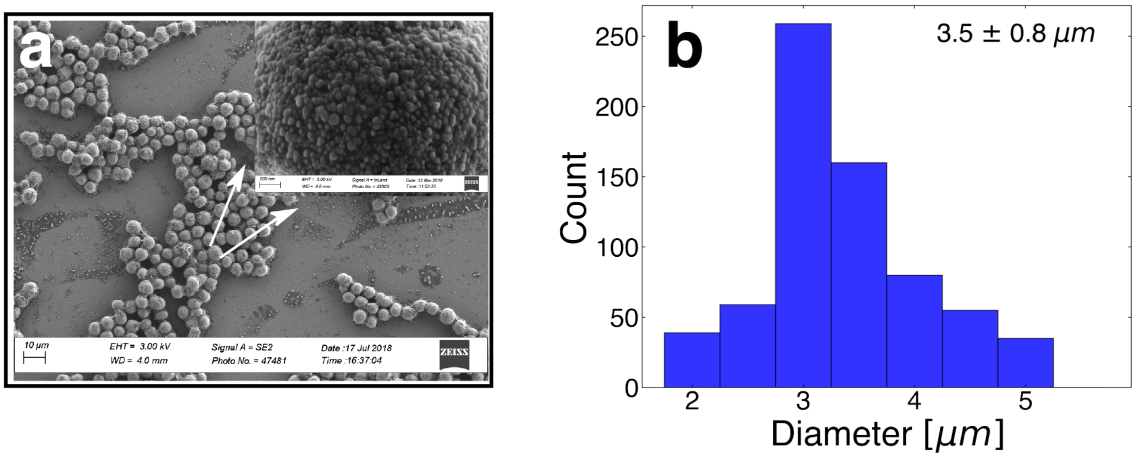

Supplementary Figure 3: **Characterization of synthesized vaterite micro-spheres.** **a** SEM micrograph of vaterite microspheres. Inset shows the poly-crystalline structure of the particle's surface. **b** Size distribution of vaterite microspheres.

Supplementary Note 6: Measuring the frequency and phase of a rotating particle

When spinning, the angle θ a vaterite particle's optical axis makes with incident linearly polarized light varies periodically so that a particle's rotation frequency $f \propto \dot{\theta}$. Throughout one period, vaterite particles depolarize the transmitted light from the polarized LED source four times. The observed depolarizations, or "blinks", occur whenever the optical axis of the particle coincides with neither the axis of the polarizer nor the analyzer (Fig. 1c). Tracking the transmitted light intensities of individual particles as a function of time, $I_i(t) = I_0 \sin^2(2f_i t + \Phi_i)$, allows for a direct measure of each particles' rotation frequency and phase, Φ_i. We normalized all signals so that $I_0 = 1$.

To measure Φ_i and f_i of rotating particles, we compute the Fourier transform of their transmitted light

intensities

$$\mathcal{F}_i = \mathcal{F}_i\left[I_i\left(t\right)\right]\left(\omega\right) \equiv \int dt e^{-\mathrm{i}\omega t} I_i\left(t\right) \\ \propto -I_0 e^{2\mathrm{i}\Phi_i}\delta\left(\omega - 4f\right), \tag{5}$$

where we have assumed that ω, Φ_i, f are positive quantities. Eq. 5 allows us to compare the phases of multiple particles globally. For individual particles, the magnitude of the Fourier transform $\sqrt{\mathcal{F}_i\mathcal{F}_i^*} \propto \delta\left(\omega - 4f\right)$. However, the sum of N-individual Fourier transforms, $F_N = \left|\frac{1}{N}\sum_i^N \mathcal{F}_i\right|^2$, decays with the number of particles. Explicitly,

$$\begin{aligned} F_N &= \left|\frac{1}{N}\sum_i^N \mathcal{F}_i\left[I_i\left(t\right)\right]\left(\omega\right)\right|^2 \\ &= \left|\frac{1}{N}\sum_i^N \int dt e^{-i\omega t} I_i\left(t\right)\right|^2 \\ &\propto \frac{I_0^2}{N^2}\delta^2\left(\omega - 4f\right)\left[N + \sum_i^N\sum_k^N e^{2i\left(\Phi_i - \Phi_k\right)}\right]. \end{aligned} \tag{6}$$

As an example, let us consider the case of $N = 2$ particles, Eq. 6 becomes,

$$F_N = \frac{I_0^2}{4}\delta^2\left(\omega - 4f\right)\left(2 + e^{2i\left(\Phi_1 - \Phi_2\right)} + e^{2i\left(\Phi_2 - \Phi_1\right)}\right) \tag{7}$$

When particles have the same global phases, $\Phi_1 = \Phi_2$, $\sqrt{F_2 F_2^\star} = I_0 = 1$. When the optical axes of the particles are perfectly out of phase $\Phi_1 - \Phi_2 = \pi/2$, $\sqrt{F_2 F_2^\star} = 0$. For all other intermediate relative phases, as – is the case in Fig. 1(c,d) – the asynchronous phases of the light intensities do not add up constructively, and the amplitude of the sum of the individual Fourier transforms is reduced.

Supplementary Note 7: Flow generated by a rotating sphere near a wall in the Stokes-flow regime

Consider an isolated sphere with radius a subjected to a constant torque $\boldsymbol{\tau}^{\mathbf{0}} = 8\pi\eta a^3\boldsymbol{\Omega}_j^0$, where η is the fluid viscosity, and Ω_j^0 is the initial angular speed of the rotor in the absence of any neighboring rotating particles. The Green's function for this rotlet is[9]

$$\mathbf{G}_{ij} = \frac{\epsilon_{ijk} r_k}{r^3}$$

and the corresponding free space flow is

$$\mathbf{u_j}\left(\mathbf{r}\right) = \epsilon_{ijk}\Omega_j^0 a^3 \frac{r_k}{r^3}.$$

Here ϵ_{ijk} is the Levi-Cevita symbol with indices representing the x, y, z directions in the Cartesian coordinate system, and r is a three-dimensional (3D) vector, $|r| \equiv \sqrt{x^2 + y^2 + z^2}$, from the source of the disturbance to a point (x, y, z) in space.

In our experiments, rotors spin near the wall of a capillary. We consider a rotating sphere spinning about an axis perpendicular to a nearby plane located at $z = 0$. The sphere's center is at a height $\delta = h + a$ (see

Fig. 5 in the main text). We assume that the plane has a no-slip boundary condition, ($\mathbf{u_j}(z=0)=0$), so that we may approximate the far-field fluid flow as the superposition of two rotlets with equal and opposite torques,$\boldsymbol{\tau} = \pm\boldsymbol{\tau^0}$, located at $z = \pm\delta$, respectively (Fig.5). The resulting fluid flow is,

$$\mathbf{u}_j(r) = \Omega_j^0 a^3 \left[\left(\frac{1}{|R_+|^3} - \frac{1}{|R_-|^3} \right) (-y\hat{\mathbf{x}} + x\hat{\mathbf{y}}) \right] \tag{8}$$

Here, $|R_\pm| \equiv \left(x^2 + y^2 + (z \mp \delta)^2 \right)^{\frac{1}{2}}$ representing the distance to a point (x, y, z) in space from the source and image charges, respectively. Next, we consider specifically the far-field limit ($\delta \ll r$) where $|R_\pm| = \left[(r^2 \left(1 \mp \frac{2\delta z}{r^2}\right) \right]^{\frac{1}{2}}$ and $|R_\pm|^{-3} \approx \frac{1}{r^3}\left(1 \pm \frac{3\delta^2}{r^2}\right)$. In the final equality, we make the approximation that the flow is strictly two-dimensional (2D) and confined to the special symmetry plane located approximately one particle radius from the wall at $z = \delta$. Eq.8 reduces to:

$$\mathbf{u}_j(r) = \frac{6\Omega_j^0 a^3 \delta^2}{r^4} \hat{\boldsymbol{\theta}}. \tag{9}$$

Note that the r^{-4} scaling is a result of the conversion from Cartesian (with basis vector $\hat{\mathbf{x}}, \hat{\mathbf{y}}$) to polar coordinates (with basis vectors $\hat{\mathbf{r}}, \hat{\boldsymbol{\theta}}$). The polar component of the flow, $\hat{\boldsymbol{\theta}}$, has been rewritten using $-r\sin\theta\hat{\mathbf{x}} + r\cos\theta\hat{\mathbf{y}} = -y\hat{\mathbf{x}} + x\hat{\mathbf{y}} = r\hat{\boldsymbol{\theta}}$. In Eq. 9, r is now the distance in the 2D plane.

Faxen's first law[10], $\mathbf{F}_i = 6\pi\eta a \left\{ \left[\mathbf{u}_j(\mathbf{r}) + \frac{1}{6} a^2 \nabla^2 \mathbf{u}_j(\mathbf{r}) \right]_{\mathbf{r}=\mathbf{r}_i} - \mathbf{v}_i \right\}$, in the presence of no external forces ($\mathbf{F}_i = 0$) reduces to

$$\mathbf{v}_i = \frac{d\mathbf{r}_i}{dt} = \mathbf{u}_j(\mathbf{r}) + \mathcal{O}\left(\frac{1}{r^6}\right) \tag{10}$$

Eq. 10 describes the tangential velocity of particle i resulting from the advective flow field generated by particle j. The modification of particle i's angular velocity (spinning rate) due to the vorticity of j's flow is found by application of Faxen's second law[10],

$$\boldsymbol{\Delta\Omega}_i \equiv \boldsymbol{\Omega}_i - \boldsymbol{\Omega}_i^0 = \frac{1}{2} \nabla \times \mathbf{u}_j(\mathbf{r} = \mathbf{r}_i). \tag{11}$$

Rewriting Eq.9 in terms of a general flow power law, α and prefactor, A, $\mathbf{u}_j(r) = Ar^{-\alpha}\Omega_j^0 \hat{\boldsymbol{\theta}}$. We compute the curl as $(\nabla \times \mathbf{u}_j) = \frac{1}{r}\left(\frac{\partial}{\partial r}\left[Ar \cdot r^{\alpha}\Omega_j^0\right]\right) = \mathbf{u}_j(r)(1-\alpha)/r$. Considering the case where rotors are of equal size, we arrive at

$$\boldsymbol{\Delta\Omega} = \frac{1-\alpha}{4} \boldsymbol{\omega}, \tag{12}$$

where we have rewritten the expression in terms of the orbital frequency of the rotating pair about their common center by using $\boldsymbol{\omega} = 2\omega_i = 2\mathbf{u}_j(r)/r$. For the case of a particle near a wall, $\alpha = 4$ and Eq.12 reduces to $\boldsymbol{\Delta\Omega} = -3\boldsymbol{\omega}/4$.

A: Measuring the change in the instantaneous spinning rate $\Delta\Omega$ of a rotor

We record videos of hydrodynamically-coupled rotors and track their trajectories and transmitted light intensities $I_i(t)$ using TrackPy[3]. For each rotor pair, local maxima and minima in $I_i(t)$ are found based on a topographic prominence algorithm distributed through the *SciPy* library. We compute the difference between

neighboring minima and maxima in $I_i(t)$, proportional to 1/8 of the particle rotation frequency. For a given window, the instantaneous Ω is found from the mean and standard error of the minima-maxima difference. The window is then advanced by a 1/8 of a rotation period, and this procedure is repeated until the entire signal is evaluated.

B: Expected fluctuations in the extracted spin rate for measuring the spin-orbit coupling

The accuracy of measuring the instantaneous rotation rate of a Brownian rotor is limited by the time over which the orientation rate is tracked. An active rotor that follows the overdamped Langevin equation $\dot{\theta} = \Omega_0 + \eta(t)$ (where Ω_0 is the nominal spinning rate, and $\eta(t)$ is the thermal noise term), follows the average dynamic analogs to a Brownian particle undergoing thermal diffusion in 1D:

$$\begin{aligned} \langle \Delta\theta \rangle &= \Omega_0 t \\ \langle \Delta\theta^2 \rangle &= 2D_r t. \end{aligned} \tag{13}$$

The mean evolution of the orientation ($\Delta\theta \equiv \theta(t) - \theta(0)$) has a drift-like term ($\langle \Delta\theta \rangle^2 \propto (\Omega_0 t)^2$). When tracking the change in orientation over some finite time, τ, the instantaneous spinning rate can be approximated by the mean $\langle \Omega_0 \rangle \approx \langle \Delta\theta \rangle / \tau$. Measurement over a finite time will also be subjected to thermal fluctuations. Hence, the measured instantaneous spinning rate will have a finite uncertainty: $\langle \Delta\Omega_0 \rangle \approx \sqrt{\langle \Delta\theta^2 \rangle}/\tau \approx \sqrt{2D_r/\tau}$. Therefore there is an intrinsic uncertainty for the measured spinning rate when evaluated over a finite period of time as done in an experiment. The relative error decreases with time as: $\mathrm{RE} = \frac{\langle \Delta\Omega_0 \rangle}{\langle \Omega_0 \rangle} = \frac{\sqrt{2D_r}}{\Omega_0 \sqrt{\tau}}$.

When two optical rotors interact, they make a transient orbit until they diffuse apart. We evaluated the instantaneous spinning rate over the duration τ_B — the time between two blinking events (see Fig. 1c in the main text). We can now compute the uncertainty in the spinning rate for a typical 4 μm particle using the empirically found parameters: $D_r \approx 0.02$ rad^2/s, $\Omega_0 \approx 1$ rad/s, $\tau_B \approx 2$ s (see Fig. 1c, Fig 3c, and Fig. 4d in the main text). This gives a relative error of $\mathrm{RE} \approx 0.14$. Compared with the nominal spinning rate, the relative error is not very large. But when compared with $\Delta\Omega$ – that is the *change* in the spinning rate (originating from the hydrodynamic spin-orbit coupling) – this thermal fluctuation is more significant. We measure a relative change in spinning rate $\Delta\Omega/\Omega_0 \lessapprox 0.3$. This value is consistent with previously theoretically predicted value[11]. As can be seen in Fig. 6d, the thermal fluctuations are small (but compatible) relative to the measured spin-orbit coupling.

References

[1] Berne, B. J. & Pecora, R. *Dynamic light scattering: With applications to chemistry, biology and physics* (Dover Publications, Inc., 2000).

[2] Lisicki, M., Cichocki, B., Rogers, S. A., Dhont, J. K. G. & Lang, P. R. Translational and rotational

near-wall diffusion of spherical colloids studied by evanescent wave scattering. *Soft Matter* **10**, 4312 (2014).

[3] Allan, D., Caswell, T., Keim, N. & van der Wel, C. trackpy: Trackpy v0.3.2 (2016). URL `https://doi.org/10.5281/zenodo.60550`.

[4] Crocker, J. C. & Grier, D. G. Methods of Digital Video Microscopy for Colloidal Studies. *J. Colloid Interface Sci.* **179**, 298–310 (1996).

[5] Arita, Y. *et al.* Rotational dynamics and heating of trapped nanovaterite particles. *ACS Nano* **10**, 11505–11510 (2016).

[6] Ashkin, A. Acceleration and trapping of particles by radiation pressure. *Phys. Rev. Lett.* **24**, 156–159 (1970).

[7] Friese, M. E. J., Nieminen, T. A., Heckenberg, N. R. & Rubinsztein-Dunlop, H. Optical alignment and spinning of laser-trapped microscopic particles. *Nature* **394**, 348–350 (1998).

[8] Vaippully, R., Gummaluri, V. S., Vijayan, C. & Roy, B. Validity of cylindrical approximation for spherical birefringent microparticles in rotational optical tweezers. *J. Phys. Commun.* **4**, 015005 (2020).

[9] Blake, J. R. & Chwang, A. T. Fundamental singularities of viscous flow. *J. Eng. Math.* **8**, 23–29 (1974).

[10] Happel, J. & Brenner, H. *Low Reynolds number hydrodynamics*. Mechanics of Fluids and Transport Processes (Kluwer Academic, Dordrecht, Netherlands, 1983).

[11] Davis, M. H. The slow translation and rotation of two unequal spheres in a viscous fluid. *Chem. Eng. Sci.* **24**, 1769–1776 (1969).
